# Clinicopathological patterns and challenges of management of colorectal cancer in a resource-limited setting: a Tanzanian experience

**DOI:** 10.1186/1477-7819-11-88

**Published:** 2013-04-18

**Authors:** Phillipo L Chalya, Mabula D Mchembe, Joseph B Mabula, Peter F Rambau, Hyasinta Jaka, Mheta Koy, Eliasa Mkongo, Nestory Masalu

**Affiliations:** 1 Department of Surgery, Catholic University of Health and Allied Sciences-Bugando, Mwanza, Tanzania; 2 Department of Surgery, Muhimbili University of Health and Allied Sciences, Dar Es Salaam, Tanzania; 3 Department of Pathology, Catholic University of Health and Allied Sciences-Bugando, Mwanza, Tanzania; 4 Department of Internal Medicine, Catholic University of Health and Allied Sciences-Bugando, Mwanza, Tanzania; 5 Department of Surgery, Amana Hospital and Hubert Kairuki Memorial University, Dar Es Salaam, Tanzania; 6 Department of Oncology, Catholic University of Health and Allied Sciences-Bugando, Mwanza, Tanzania

**Keywords:** Colorectal cancer, Clinicopathological, Challenges, Resource-limited setting, Tanzania

## Abstract

**Background:**

Colorectal cancer is one of the most common cancers worldwide and its incidence is reported to be increasing in resource-limited countries, probably due to the acquisition of a western lifestyle. However, information regarding colorectal cancer in Tanzania and the study area in particular is limited. This study was conducted in our local setting to describe the clinicopathological pattern of colorectal cancer and highlight the challenging problem in the management of this disease.

**Methods:**

This was a retrospective study of histologically confirmed cases of colorectal cancer seen at Bugando Medical Center between July 2006 and June 2011. Data were retrieved from patients’ files and analyzed using SPSS computer software version 17.0.

**Results:**

A total of 332 colorectal cancer patients were enrolled in the study, representing 4.7% of all malignancies. Males outnumbered females by a ratio of 1.6:1. The median age of patients at presentation was 46 years. The majority of patients (96.7%) presented late with advanced stages. Lymph node and distant metastasis at the time of diagnosis was recorded in 30.4% and 24.7% of cases, respectively. The rectosigmoid region was the most frequent anatomical site (54.8%) involved and adenocarcinoma (98.8%) was the most common histopathological type. The majority of adenocarcinomas (56.4%) were moderately differentiated. Mucinous and signet ring carcinomas accounted for 38 (11.6%)and 15 (4.6%) patients, respectively. Three hundred and twenty-six (98.2%) patients underwent surgical procedures for colorectal cancer. Only 54 out of 321 (16.8%) patients received adjuvant treatment. Postoperative complication and mortality rates were 26.2% and 10.5%, respectively. The overall median duration of hospital stay was 12 days. Only nine out of 297 survivors (3.0%) were available for follow-up at the end of 5 years. Cancer recurrence was reported in 56 of 297 survivors (18.9%). Data on long-term survival were not available as the majority of patients were lost to follow-up.

**Conclusions:**

Colorectal cancer is not uncommon in our environment and shows a trend towards a relative young age at diagnosis and the majority of patients present late with advanced stage. There is a need for screening of high-risk populations, early diagnosis and effective cost-effective treatment and follow-up to improve outcome of these patients.

## Background

Colorectal cancer constitutes a major public health issue globally with an estimated 1.2 million new cancer cases and over 630,000 cancer deaths per year, almost 8% of all cancer deaths [1,2]. It is the fourth most common cancer in men and the third most common in women worldwide [2]. Globally, the incidence of colorectal cancer varies widely by over 10-fold, with the highest incidence rates in Australia and New Zealand, Europe and North America, and the lowest rates in Africa and Asia [1,3,4]. These geographic differences appear to be attributable to differences in dietary and environmental exposures that are imposed upon a background of genetically determined susceptibility [4]. However, despite this variation, the molecular characteristics remain similar throughout the world [5,6]. While recent studies have shown that the incidence of colorectal cancer is increasing in sub-Saharan Africa, especially in the urban centers [7,8], the converse is true in the developed world where the incidence of colorectal cancer is declining with a relatively good outcome of treatment attributable to early detection, especially in high-risk individuals due to widespread screening of the susceptible population, early presentation, and advances in diagnostic tools and treatment modalities [9].

The clinicopathological patterns of colorectal cancer have been reported to vary in different geographical regions. While in the western world colorectal cancer is a disease of older patients, with most being diagnosed after the age of 50 years [3,4,10], colorectal cancer in African population tends to present at a young age with advanced aggressive disease and associated poor prognosis [11-14].

The clinical stage of the disease at diagnosis often determines the prognosis and survival rate of a patient with colorectal cancer, with the best outcomes seen in patients diagnosed at an early stage. However, the outcome of treatment of colorectal cancer in our environment has been poor because the majority of these patients present late to the hospital with advanced stage and only palliative care is possible [10-14]. This is partly due to paucity of local data regarding this condition and lack of community awareness on the importance of early reporting to hospital for early diagnosis and treatment.

Colorectal cancer and its treatment constitute a great diagnostic and therapeutic challenge in resource-limited countries such as Tanzania. Late presentation of the disease, lack of an adequate screening program, lack of endoscopic facilities, lack of adjuvant therapy and a high morbidity and mortality are among the hallmarks of the disease in these countries [5,11-15].

The current treatment options of colorectal cancer consist of surgery for early-stage disease and the combination of surgery and adjuvant therapy for advanced-stage disease [16]. Surgery is currently the only golden standard treatment for colorectal cancer with curative potential [16,17]. However, curative surgery in most developing countries is limited due to the late presentation in the majority of patients. Lack of adjuvant therapy in most centers also poses a therapeutic challenge in resource-limited countries.

Colorectal cancer is one of the cancers that can be prevented by secondary prevention, which can be achieved by screening tests that can detect pre-neoplastic lesions and early cancer. Colorectal cancer screening programs increase the detection of tumors at earlier stages and reduce colorectal cancer incidence and mortality [18]. In resource-limited countries, however, lack of a screening program poses a great challenge in the prevention of colorectal cancer.

There is a paucity of information regarding colorectal cancer in Tanzania and particularly the study area. This is partly due to a lack of published local data regarding this condition and lack of cancer registries in this region. This study was designed to describe the clinicopathological pattern of colorectal cancer and highlight the challenging problem in the management of this disease in our local setting.

## Methods

### Study design and setting

Between July 2006 and June 2011, a retrospective study of histologically confirmed cases of colorectal cancer was conducted at Bugando Medical Center. Bugando Medical Center is a consultant and teaching hospital for the Catholic University of Health and Allied Sciences-Bugando (CUHAS-Bugando) in the Lake and Western Zones of the United Republic of Tanzania. It is situated along the shores of Lake Victoria in Mwanza City. It has 1000 beds and serves as a referral center for tertiary specialist care for a catchment population of approximately 13 million people. The hospital has a newly established oncology department which provides care for all patients with histopathologically proven cancers, including colorectal cancers. However, the department does not provide radiotherapy services at the moment due to lack of this facility at our center. As a result, patients requiring this modality of treatment have to travel long distances to receive radiotherapy at the Tanzania Tumor Center located a considerable distance from the study area.

### Study population

The subjects of this study included all patients who presented to Bugando Medical Centerwith histologically confirmed colorectal cancer during the study period. Patients with incomplete data were excluded from the study.

The details of patients were retrieved from patients’ files kept in the medical record department, the surgical wards, operating theatre and histopathology laboratory. Information retrieved included socio-demographic data, clinical presentation, anatomical site, gross appearance, tumor stage, histopathological type and grade, presence of metastasis (nodal, distant and peritoneal), treatment modalities and outcome and follow-up. The anatomical site distributions were categorized as right colonic, left colonic and rectal tumors. The tumor stage was ascertained using the Tumor-Node-Metastases (TNM) staging system of the International Union against Cancer.

The diagnosis of colorectal cancer was performed by barium enema, proctosigmoidoscopy and laparotomy and confirmed histopathologically by endoscopic and laparotomy biopsies. Baseline hemogram and stool microscopy, proctosigmoidoscopy, abdominal ultrasound, liver function tests and chest radiographs were performed routinely. Barium enema was performed only in some patients. Carcinoembryonic antigen level was not recorded in any patient. Colonoscopic examination was also not performed due to its nonavailability. Treatment modalities included surgery and adjuvant chemotherapy and radiotherapy. Patients were followed up for up to 5 years or death. Survival analysis was carried out, with survival defined as the time between the date of commencement of treatment and the date of last follow-up or death. The recurrence of disease was confirmed by physical findings, radiological studies, endoscopic examination with biopsy, and surgery.

### Definitions of variables

*Right-sided:* Lesions arising from the cecum, ascending colon, hepatic flexure or transverse colon.

*Left-sided:* Lesions arising from splenic flexure, descending colon or sigmoid colon.

*Dukes’ A:* Growth confined to the wall

*Dukes’B:* Growth to the serosa and beyond, but no nodal involvement

*Dukes’ C:* Growth beyond serosa and involving nodal basins

*Dukes’D:* Metastatic disease

### Statistical analysis

Data collected were analyzed using SPSS computer software version 17.0 (SPSS, Inc., Chicago, IL, USA). Data were summarized in the form of proportions and frequency tables for categorical variables. Continuous variables were summarized using mean, median, mode and standard deviation. Chi-square test was used to test for significance of associations between the predictor and outcome variables in the categorical variables. Student’s t test was used to test for significance of associations between the predictor and outcome variables in the continuous variables. Significance was defined as a *P*value of <0.05. Multivariate logistic regression analysis was used to determine predictor variables that are associated with outcome.

### Ethical consideration

Ethical approval to conduct the study was sought from the CUHAS-Bugando/Bugando Medical Center joint institutional ethic review committee before the commencement of the study.

## Results

### Study population and demographic data

During the study period, a total of 7014 malignancies were registered. Of these, 332 (4.7%) were histopathologically confirmed colorectal cancer which formed the study population. The number of males was 202 (60.8%) and the number of females was 130 (39.2%) with a male to female ratio of 1.6:1. The age ranged from 15 to 82 years with a median age of 46 years. The modal age group at presentation was 41–50 years. One hundred and twenty-seven (38.3%) patients were aged 40 years and below. Family history of colorectal cancer was reported in 18 (5.4%) cases. The majority of patients, 234(70.5%) came from the rural areas located a considerable distance from Mwanza City and most of them, 204(61.4%), had either primary or no formal education.

### Clinical presentation, anatomical sites, gross appearance, histological patterns and tumor stage

The duration of symptoms at presentation ranged from 3 weeks to 7 years with a median duration of 22 months. Two hundred and forty-two (72.9%) patients presented within 6 months of onset and 90 (27.1%) presented longer than 6 months. Table 1 shows clinical presentation of colorectal cancer patients.

**Table 1:** Distribution of patients according to clinical presentation

**Clinical presentation**	**Frequency**	**Percentage**
Rectal bleeding	180	54.2
Change in bowel habit	172	51.8
Abdominal pain	153	46.1
Constipation	140	42.2
Weight loss	122	36.7
Anemia	98	29.5
Abdominal mass	56	16.9
Feeling of incomplete evacuation	34	10.2
Anal mass	30	9.0

There were 202 (60.8%) left-sided (distal colon) tumors, 78 (23.5%) right-sided (proximal) tumors and 52 (15.7%) rectal tumors. The recto-sigmoid region was the most frequent site for colorectal cancer in 182 (54.8%) cases followed by cecum, ascending colon, descending colon and transverse colon in 40 (12.0%), 25 (7.5%), 20 (6.0%) and13 (3.9%) cases, respectively. The anorectum was involved in 52 (15.7%) cases. Of these, the tumor was palpable on digital rectal examination in 48 (92.3%) cases and in the remaining 4 (7.7%) patients the tumor was accessible only by proctoscopic and sigmoidoscopic examinations. There was no recorded cases of synchronous or metachronous malignancies.

Macroscopically, the right-sided tumors were fungating nodular lesions with surface ulcerations while the left-sided tumors were flat and infiltrating or constricting.

Microscopically, adenocarcinoma was the most common histopathological tumor in 328 (98.8%) patients. The majority of adenocarcinomas were moderately differentiated adenocarcinoma in 185(56.4%), 102 (31.1%) were well-differentiated and 41(12.5%) were poorly differentiated carcinoma. Mucinous and signet ring carcinomas accounted for 38 (11.6%) and 15 (4.6%) patients, respectively. Seventeen (5.2%) were anaplastic (undifferentiated) tumors. Mucinous carcinoma (9.1%) and signet ring carcinoma (3.4%) were statistically significantly more common in patients under 40 years, compared to 2.4% and 1.2% recorded in patients above 40 years, respectively (*P*< 0.001). Mucinous adenocarcinomas were statistically significantly more frequent in right-sided (proximal) colon (9.8%) than in left-sided colon (1.8%) (*P* =0.011). Mucinous adenocarcinomas and signet ring carcinoma were significantly associated with a poor prognosis compared to non- mucinous adenocarcinomas (*P*< 0.001). Other histopathological tumors were non-Hodgkin’s lymphoma, malignant gastrointestinal stromal tumors, lieomyosarcoma and squamous cell carcinoma in 1 (0.3%) patient each, respectively.

According to TNM staging, only 11 (3.3%) patients were identified as being in early stages (TNM stage I) and 321 (96.7%) patients were presented in advanced stages (stage II-IV): Table 2 shows pathological staging according to TNM staging. There were no statistically significant differences between the right-sided and left-sided colonic carcinoma regarding sex, age, histological grade and tumor stage (*P*> 0.001). Lymph node metastasis at the time of diagnosis was recorded in101 (30.4%) cases. Distant metastasis was recorded in 82 (24.7%) cases.

**Table 2:** Distribution of patients according to pathological stage

**TNM stage**	**N/%**
I	11 (3.3)
II	138 (41.6)
III	101(30.4)
IV	82 (24.7)

### Diagnosis of colorectal cancer

The diagnosis of colorectal cancer was made by barium enema and proctosigmoidoscopy in 111 (33.4%) and 98 (29.5%) patients, respectively. The remaining 123 (37.0%) patients were diagnosed during laparotomy for other pathologies such as abdominal mass in 68 (55.3%) patients, intestinal obstruction in 38(30.9%), intussusception in 10(8.1%) and bowel perforation in 6 (4.9%) patients. No patients had colonoscopic examination.

### Treatment modalities

Out of 332 patients, 326 (98.2%) patients underwent surgical procedures for colorectal cancer and the remaining 6 (1.8%) patients were unfit for surgery. Of these 326, 282 (86.5%) patients were operated electively and the remaining 44 (13.5%) were operated on an emergency basis for intestinal obstruction in 38 (86.4%) and bowel perforation with peritonitis in 6 (13.6%) patients. Left hemicolectomy was the most frequent type of surgical procedure performed, accounting for 58.6% of cases (Table 3).

**Table 3:** Distribution of patients according to the type of surgical procedures performed (N= 326)

**Surgical procedures performed**	**Frequency**	**Percentage**
Left hemicolectomy	191	58.6
Right hemicolectomy	72	22.1
Abdomino-perineal resection	28	8.6
Transverse colectomy	12	3.7
Exploratory laparotomy + biopsy only	10	3.1
Anterior resection	8	2.5
Colostomy alone	5	1.5

Adjuvant treatment (radiation therapy and/or chemotherapy) was indicated in 321 (96.7%) patients. Of these, only 54 (16.8%) received adjuvant treatment. Out of 54 patients, 30 (9.3%) received chemotherapy, 23 (7.2%) received radiotherapy and only 1 (0.3%) patient received chemo-radiation therapy.

### Treatment outcome and follow-up of patients

Postoperative complications were recorded in 86 (26.2%) patients. Of these, surgical site infection was the most common postoperative complication accounting for 41.9% of cases (Table 4). A total of 18 (5.5%) 5.5% = 18/326 and not 18/321. patients required additional operation for perineal wound breakdown (8 patients)), intra-abdominal abscess/peritonitis (8 patients) and anastomotic breakdown (2 patients).

**Table 4:** Distribution of patients according to postoperative complications (N = 86)

**Postoperative complications**	**Frequency**	**Percentage**
Surgical site infections	36	41.9
Perianal wound breakdown	11	12.8
Intra-abdominal abscess/peritonitis	8	9.3
Anastomotic breakdown	5	5.8
Chest infections	5	5.8
Anemia	4	4.7
Septicemia	4	4.7
Renal failure	2	2.3
Deep venous thrombosis	1	1.2

The overall hospital stay ranged between 4 and 72 days with a median of 12 days. Patients who developed postoperative complications and those who had abdomino-perineal resection had a longer hospital stay (*P*< 0.001).

Out of 332 patients, 297 (89.5%) were alive. Thirty-five patients died in hospital giving a mortality rate of 10.5%. Of those who were alive, 264(88.9%) were discharged well and the remaining 33 (11.1%) were discharged with permanent colostomy.

Follow-up of patients among survivors ranged from 3 to 61 months with a median of 18 months. At the end of 3 months, 6 months, 2 years and 5 years, 45(15.2%), 34 (11.4%), 15 (5.1%) and 9 (3.0%) patients (survivors), respectively, were available for follow-up. Cancer recurrence was reported in 56 (18.9%) patients. Data on long-term survivals were not available as the majority of patients were lost to follow-up.

## Discussion

In this review, colorectal cancer accounted for 4.7% of all diagnosed malignancies seen during the study period in our setting. This concurs with figures of 3.7 to 10% that have been reported from various parts of Africa [15,17,19-21]. There is a marked variation in the incidence of colorectal cancer worldwide, with western countries having a high rate compared to Africa [1,3,4]. However, a rising incidence of colorectal cancer has been reported from various parts of Africa which were considered low incidence areas [11-13,20]. This rising incidence of colorectal cancer in Africa has been attributed to improved cancer awareness and a shift towards a western diet.

As reported in other African studies [11-13], the median age of 46 years in this study was younger than the age described in most developed countries; about 10 years difference has been reported in these studies. In this study, 38.3% of colorectal cancer patients were found to be below the age of 40 years, a figure which is higher than the figure of 2 to 6% reported in western countries [22,23]. Higher figures of colorectal cancer patients aged below the age of 40 years were also reported in other studies [5,12]. In the African population, colorectal cancer tends to be more aggressive and occurs at a relatively young age at the time of presentation. Colorectal cancer in the younger age group has been shown to present a diagnostic and therapeutic problem and prognosis tends to be less favorable [14]. The presence of a high number of young patients with colorectal cancer in low-risk communities necessitates family screening since genetic factors may play an important role in the development of this disease. This makes early detection and management an important measure in order to reduce incidence and mortality.

The male predominance demonstrated in this study was in keeping with previous observations reported in studies performed elsewhere [11,24-26]. We could not establish the reason for the male predominance.

In this study, a family history of colorectal cancer was reported in 5.4% of cases, a figure which is slightly higher than 4.3% reported by Azadeh *et al.*[27] in Iran. This suggests that genetic factors may be playing an important role in the development of this disease in our country. Based on this alarming observation we suggest that screening programs, especially genetic screening programs, should be considered as a main measure for prevention and control of colorectal cancer in this part of the world.

In keeping with other studies performed in developing countries [12,15,17,21,24,26], the majority of patients in this series came from the rural areas located a considerable distance from the study area and more than 60% had either primary or no formal education. This observation has an implication on accessibility to healthcare facilities and awareness of the disease.

In the present study, the majority of patients presented late with advanced stage of cancer which is in keeping with other studies in developing countries [5,11-15,28-30]. Late presentation in our study may be attributed to lack of awareness of the disease, low standard of education, lack of accessibility to healthcare facilities and lack of screening programs in this region. Late presentation of cases is an area of colorectal cancer care in our center that requires urgent attention. There needs to be an increased awareness of the symptoms of colorectal cancer in the general population, along with implementation of screening programs in this region. Detecting colorectal cancer at an early stage contributes to improved chances for successful treatment and thus for survival.

Rectal bleeding was the commonest symptom for colorectal cancer in the current study which is in agreement with other studies reported in developing countries [31,32]. Bleeding per rectum is a common problem that prompts patients to seek medical help. It remains a diagnostic challenge, on the basis of bleeding alone, to distinguish a benign anal condition from a serious underlying colorectal disease. Bleeding is the index symptom in early stage colorectal cancer and merits urgent and full investigation [31]. High rectal bleeding rate among patients with colorectal cancer in the present study may be attributed to the large number of patients with left-sided tumors, particularly the rectosigmoid and anorectal tumors. Previous studies have, however, indicated that rectal bleeding can reasonably predict colorectal cancer, especially when reported in older patients and in combination with a change in bowel habits or abdominal pain [33,34].It would appear, therefore, that it may be worth investigating rectal bleeding in all cases. Early colorectal investigations for the patients with rectal bleeding, change of bowel habits and anemia may improve the outlook.

Regarding the anatomical location of colorectal cancer at presentation, there was a preponderance of left-sided tumors, especially rectosigmoid, in this study as reported by others in developing countries [12-14,31,32,35,36]. This contrasts with the right-side preponderance (proximal shift) reported in developed countries [37,38]. The reason for this anatomical difference among these countries is not clear.

Macroscopically, the left-sided tumors were flat and infiltrating or constricting whereas the right-sided tumors were fungating nodular lesions with surface ulcerations. This observation concurs with Abdulkareem *et al.*[39] in Nigeria. Tumors on the right side (ascending colon and cecum) tend to be exophytic; that is, the tumor grows outwards from one location in the bowel wall. This very rarely causes obstruction of feces, and presents with symptoms such as anemia. Left-sided tumors tend to be circumferential, and can obstruct the bowel much like a napkin ring [39].

Microscopically, approximately 95% of colorectal cancers are adenocarcinomas arising from the epithelial lining of the colorectum [40]. In this study, adenocarcinoma was the most frequent (98.8%) with moderately differentiated tumors accounting for more than 50% of cases. This finding is in agreement with Missaoui *et al.*[41] in Tunisia who reported similar histopathological patterns. Other histopathological tumors in the present study were non-Hodgkin’s lymphoma, malignant gastrointestinal stromal tumors, lieomyosarcoma and squamous cell carcinoma. The present study also showed that patients <40 years of age tended to have poor prognostic tumors such as mucinous and signet ring carcinoma. This finding concurs with Fazeli *et al.*[25] in Iran who reported that 22% of patients less than 40 years of age had poorly differentiated tumor compared to 5.9% in patients above 40 years of age. Mucinous carcinomas have been associated with poor prognosis with poor response to chemotherapy and tend to be located in the proximal colon and associated with microsatellite instability [42].

According to TNM staging, more than 90% of patients in this series presented with advanced stages and only 3.3% of cases presented with early-stage cancer. This finding concurs with most reports from Africa [15,25,26,43,44]. This can be attributed mainly to the absence of specific screening measures for colorectal cancer among the Tanzanian population. There is considerable evidence that screening of asymptomatic persons who are at average risk can detect cancers at an early and curable stage, resulting in a reduction in mortality [18]. Furthermore, some screening tests may also detect cancer-precursor lesions, which, if removed, may result in a reduced incidence of colorectal cancer [45].

In this study, lymph node and distant metastases at the time of diagnosis were recorded in 30.4% and 24.7% of cases respectively. A similar metastatic pattern was reported by Yawe *et al.*[32] in Nigeria. High lymph node and distant metastases in this study is attributed to the late presentation in the majority of patients and this confirmed the highly metastatic potential of colorectal cancer.

The diagnosis of colorectal cancer in our series was made on the basis of digital rectal examination, barium enema and proctosigmoidoscopy, and the majority of patients were diagnosed during laparotomy for other pathologies such as abdominal mass, intestinal obstruction, intussusception and bowel perforation. In this study, no patient had colonoscopy due to the lack of this facility at our center. A similar diagnostic pattern was reported in most African centers [15,25,26,32]. Lack of colonoscopy at our center remains a major challenge in the management of colorectal cancer as early recognition of symptomatic colorectal cancer can be a challenge for the physician, resulting in a delay in the start of appropriate treatment.

The treatment of colorectal cancer requires a multidisciplinary approach. Treatment modalities of colorectal cancer include surgery combined with chemotherapy and radiotherapy given either as neo or adjuvant therapy [16]. Surgery continues to be the primary treatment option for colorectal cancer patients and colon/rectal resection has been the standard procedure for cancer primarily localized in the colon/rectum [16,17]. Complete resection of colorectal cancer with resection of adjacent lymph node is the only chance for cure in early-stage cancer [16,17,32]. However, most of the patients we see in our environment present late with advanced disease at the time of diagnosis, for which only palliative surgery is possible. In this study, only 3.3% of patients had colorectal cancer resection with curative intent which is in agreement with other studies in developing countries [15,25,26,43,44]. The low resection rate of colorectal cancer with “curative” intent in our series could be explained by the high proportion of patients with advanced colorectal cancer at presentation. Although anorectal cancer was diagnosed in 52 patients, only 28 (50%) patients had abdomino-perineal resection and end-colostomy. Five patients with advanced anorectal cancer had colostomy alone. Sphincter saving surgery was not practicable as lesions were not only too low but also advanced. This observation can be explained by the fact that the majority of our patients presented late with features of advanced disease, as similarly reported by Yawe *etal.*[32]. This late presentation still poses a challenge to management of colorectal cancer in our setting and requires public education.

Despite the fact that surgery is the main stay of treatment, radiotherapy and chemotherapy play a vital role, particularly in locally advanced colorectal tumor [16]. The majority of patients with colorectal cancer in this study had advanced stage of the disease at presentation and were therefore candidates for this form of treatment. In the present study, only 9.3% of patients received chemotherapy despite the establishment of an oncological unit in our center in 2009. In resource poor countries such as Tanzania, nonadherence to chemotherapy is a major challenge in cancer treatment including colorectal cancer. Reasons for nonadherence in most developing countries include financial difficulty, resorting to alternative treatment and drug side effects [32]. We could not establish the reasons for nonadherence to chemotherapy in our study due to the retrospective nature of the study. Further prospective study is needed to explain this observation.

Radiation therapy has been shown to improve local control rates in locally advanced colorectal cancers when applied preoperatively or postoperatively. However, in our study only 7.2% of patients who required radiotherapy had access to this form of treatment. This concurs with other studies in resourcelimited countries [32,46]. Failure to access this modality of treatment in our patients can be explained by the fact that radiotherapy is not available in our center and therefore patients requiring this form of treatment had to travel long distances to receive radiotherapy at the oncological center. This sad observation calls for urgent establishment of radiotherapy services in our center.

In this study, postoperative complications were recorded in 26.2% which is higher than that of 12.6% reported by Datubo *et al.*[47] in Ghana. The higher complication rate in this study is attributed to late presentation and delayed definitive treatment. Additional operations were required in 5.5% of patients who developed postoperative complications such as perianal wound breakdown, intra-abdominal abscesses, peritonitis and anastomotic leak. These required additional operations that are associated with a prolonged hospital stay and cost of treatment. An improvement in surgical technique is therefore crucial to lower the occurrence of these postoperative complications while their prompt recognition and treatment would reduce the attendant mortality.

The median duration of hospital stay in our study was 12 days, which is higher than that reported in other studies [32,47]. This can be explained by a high rate of postoperative complications which required additional operations/care and subsequently prolonged hospital stay. Also, patients who had abdomino-perineal resection had a longer hospital stay. Prolonged duration of hospital stay has an impact on hospital resources as well as on increased cost of healthcare, loss of productivity and reduced quality of life.

Our overall mortality rate in the present study was 10.5%, a figure that is higher than 6.1% reported by Datubo *et al.*[47] in Ghana. The high mortality rate in our study may be attributed to the fact that most patients presented late with advanced stage.

The follow-up of patients in this study was generally poor, and data on long-term survival were not available. This observation concurs with Yawe *et al.*[32] in Nigeria. Poor follow-up of patients in our study may be explained by the fact that the majority of patients were lost to follow-up at the end of 5years.

The potential limitation of this study is the fact that information about some patients was incomplete in view of the retrospective nature of the study and this might have introduced some bias in our findings. Also, this study included only patients who were evaluated and treated at a single institution, which may not reflect the whole population in this region, despite the fact that approximately 70% of oncologic patients in northwestern Tanzania are managed at our center. However, despite this limitation, the study has provided local data that can help healthcare providers in the management of patients with colorectal cancer. The challenges identified in the management of colorectal cancer in our setting need to be addressed in order to deliver optimal care for these patients.

## Conclusions

Compared to data from western countries, colorectal cancer in this environment shows a trend towards relative young age at diagnosis and the majority of patients present late with advanced stage. Lack of awareness of the disease, poor accessibility to healthcare facilities, lack of diagnostic facilities, lack of screening programs, poor accessibility to adjuvant therapy, the high cost of care and a high morbidity and mortality are among the hallmarks of the disease in this region and pose a great challenge in the management of these patients. Therefore, public enlightenment, screening of high-risk populations, early diagnosis, and effective cost-effective treatment and follow-up will help reverse this trend.

## Abbreviations

CUHAS-Bugando: Catholic University of Health and Allied Sciences-Bugando; TNM: Tumor-Node-Metastases.

## Competing interests

The authors declare that they have no competing interests. The study had no external funding. Operational costs were met by authors.

## Authors’ contributions

PLC and HJ conceived the study and participated in the literature search, writing the manuscript and editing. In addition, HJ participated in submission of the manuscript. MDM, JBM, PFREM and NM participated in study design, data analysis, manuscript writing and editing. All the authors read and approved the final manuscript.

## References

[B1] Jemal A Bray F Center MM Ferlay J Ward E Forman D Global cancer statistics CA Cancer J Clin 2011 61 69 90 10.3322/caac.20107 21296855

[B2] Kamangar F Dores GM Anderson WF Patterns of cancer incidence, mortality, and prevalence across five continents: defining priorities to reduce cancer disparities in different geographic regions of the world J Clin Oncol 2006 24 2137 2150 10.1200/JCO.2005.05.2308 16682732

[B3] Ferlay J Bray F Pisani P Parkin D GLOBOCAN 2002: Cancer Incidence, Mortality and Prevalence Worldwide 2004 Lyon, France: IARC

[B4] Center MM Jemal A Smith RA Ward E Worldwide variations in colorectal cancer CA Cancer J Clin 2009 59 366 378 10.3322/caac.20038 19897840

[B5] Adekunle OO Abioye AA Adenocarcinoma of the large bowel in Nigerians: a clinicopathological study Dis Colon Rectum 1980 23 559 563 10.1007/BF02988996 7460693

[B6] Weitz J Koch M Debus J Hohler T Galle PR Buchler MW Colorectal cancer Lancet 2005 365 153 165 10.1016/S0140-6736(05)17706-X 15639298

[B7] Siegel R Ward E Brawley O Jemal A Cancer statistics, 2011: the impact of eliminating socioeconomic and racial disparities on premature cancer deaths CA Cancer J Clin 2011 61 212 236 10.3322/caac.20121 21685461

[B8] Soliman AS Bondy ML El-Badawy SA Mokhtar N Eissa S Bayoumy S Seifeldin IA Houlihan PS Lukish JR Watanabe T Chan AO Zhu D Amos CI Levin B Hamilton SR Contrasting molecular pathology of colorectal carcinoma in Egyptian and western patients Br J Cancer 2001 85 1037 1046 10.1054/bjoc.2001.1838 11592777 PMC2375101

[B9] Troisi RJ Freedman AN Devesa SS Incidence of colorectal carcinoma in the U.S.: an update of trends by gender, race, age, subsite, and stage, 1975–1994 Cancer 1999 85 1670 1676 10.1002/(SICI)1097-0142(19990415)85:8<1670::AID-CNCR5>3.0.CO;2-M 10223559

[B10] Boyle P Langman JS ABC of colorectal cancer epidemiology BMJ 2000 321 805 808 10.1136/bmj.321.7264.805 11009523 PMC1118620

[B11] Seleye-Fubara D Gbobo I Pathological study of colorectal carcinoma in adult Nigerians: a study of 45 cases Niger J Med 2005 14 167 172 16083240 10.4314/njm.v14i2.37175

[B12] Ojo OS Odesanmi WO Akinola OO The surgical pathology of colorectal carcinomas in Nigerians Trop Gastroenterol 1992 13 64 69 1413101

[B13] Adesanya AA da Rocha-Afodu JT Colorectal cancer in Lagos: a critical review of 100 cases Niger Postgrad Med J 2000 7 129 136 11257919

[B14] Sule AZ Mandong BM Malignant colorectal tumors in patients 30 years and below: a review of 35 cases Cent Afr J Med 1999 45 209 212 10697917 10.4314/cajm.v45i8.8486

[B15] Saidi H Nyaim EO Githaiga JW Karuri D Colorectal cancer surgery trends in Kenya, 1993–2005 World J Surg 2008 32 217 223 10.1007/s00268-007-9301-2 18057984

[B16] Cunningham D Atkin W Lenz HJ Lynch HT Minsky B Nordlinger B Starling N Colorectal cancer Lancet 2010 375 1030 1047 10.1016/S0140-6736(10)60353-4 20304247

[B17] Irabor DO Adedeji OO Colorectal cancer in Nigerians: 40 years on A review. Eur J Cancer Care 2009 18 110 115 10.1111/j.1365-2354.2008.00982.x 19267725

[B18] Walsh JM Terdiman JP Colorectal cancer screening: a scientific review JAMA 2003 289 1288 1296 10.1001/jama.289.10.1288 12633191

[B19] El Mistiri M Verdecchia A Rashid I El Sahli N El Mangush M Federico M Cancer incidence in eastern Libya: the first report from the Benghazi Cancer Registry, 2003 Int J Cancer 2007 120 392 397 10.1002/ijc.22273 17066425

[B20] Okobia MN Aligbe JU Pattern of malignant diseases at the University of Benin Teaching Hospital Trop Doct 2005 35 91 92 10.1258/0049475054037147 15970031

[B21] Kenda JF Cancer of the large bowel in the African: a 15-year survey at Kinshasa University Hospital, Zaire Br J Surg 1976 63 966 968 10.1002/bjs.1800631220 188510

[B22] Mitry E Benhamiche AM Jouve JL Clinard F Finn-Faivre C Faivre J Colorectal adenocarcinoma*in*patients under 45 years*of*age*:*comparison with older patients in a well*-*defined French population Dis Colon Rectum 2001 44 3 380 387 5 10.1007/BF02234737 11289284

[B23] Guillem JG Puig-La CalleJr J Cellini C Murray VM Ng J Fazzari M Paty PB Varying features of early age-of-onset‘sporadic’ and hereditary nonpolyposis colorectal cancerpatients Dis Colon Rectum 1999 42 36 42 10.1007/BF02235180 10211518

[B24] Edino ST Mohammed AZ Ochicha O Characteristics of colorectal carcinoma in Kano, Nigeria: an analysis of 50 cases Niger J Med 2005 14 161 166 16083239 10.4314/njm.v14i2.37174

[B25] Fazeli MS Adel MG Lebaschi AH Colorectal carcinoma: a retrospective, descriptive study of age, gender, subsite, stage, and differentiation in Iran from 1995 to 2001 as observed in Tehran University Dis Colon Rectum 2007 50 990 995 10.1007/s10350-007-0248-z 17525859

[B26] Sule AZ Mandong BM Iya D Malignant colorectal tumors: a ten year review in Jos, Nigeria West Afr J Med 2001 20 251 255 11885882

[B27] Azadeh S Moghimi-Dehkordi B Fatemi SR Pourhoseingholi MA Ghiasi S Zali MR Colorectal cancer in Iran: an epidemiological study Asian Pacific J Cancer Prev 2008 9 123 126 18439090

[B28] Asuquo M Akan I Nwagbara V Jibril P Malignant large bowel obstruction Niger J Surg Sci 2005 15 67 70

[B29] Dem A Kasse AA Diop M Gaye-Fall MC Doui A Diop PS Toure P Epidemiological and therapeutic aspects of rectal cancer; 74 cases at the cancer institute of Dakar Dakar Med 2000 45 66 69 14666795

[B30] Tade AO Right sided colon cancer at Olabisi Onabanjo University Teaching Hospital, Sagamu Nigeria; a ten-year review Nig Med Practitioner 2006 49 82 84

[B31] Saidi HS Karuri D Nyaim EO Correlation of clinical data, anatomical site and disease stage in colorectal cancer East Afr Med J 2008 85 259 262 18817021 10.4314/eamj.v85i6.9622

[B32] Yawe KT Bakari AA Pindiga UH Mayun AA Clinicopathological pattern and challenges in the management of colorectal cancer in sub-Saharan Africa J Chinese Clin Med 2007 2 688 695

[B33] Norrelund N Norrelund H Colorectal cancer and polyps in patients aged 40 years and over who consult a GP with rectal bleeding Fam Pract 1996 13 160 165 10.1093/fampra/13.2.160 8732328

[B34] Selvachandran S Hodder R Ballal M Jones P Cade D Prediction of colorectal cancer by a patient consultation questionnaire and scoring system: a prospective study Lancet 2002 360 278 283 10.1016/S0140-6736(02)09549-1 12147370

[B35] Abu-Zeid AA Khafagy W Marzouk DM Alaa A Mostafa I Ela MA Colorectal cancer in Egypt Dis Colon Rectum 2002 45 1255 1260 10.1007/s10350-004-6401-z 12352245

[B36] Kim DW Bang YJ Heo DS Kim NK Colorectal cancer in Korea: characteristics and trends Tumori 2002 88 262 265 12400972 10.1177/030089160208800402

[B37] Takada H Ohsawa T Iwamoto S Yoshida R Nakano M Imada S Yoshioka K Okuno M Masuya Y Hasegawa K Kamano H Hioki K Muto T Koyama Y Changing site distribution of colorectal cancer in Japan Dis Colon Rectum 2002 45 1249 1254 10.1007/s10350-004-6400-0 12352244

[B38] Ponz de Leon M Marino M Benatti P Rossi G Menigatti M Pedroni M Di Gregorio C Losi L Borghi F Scarselli A Ponti G Roncari B Zangardi G Abbati G Ascari E Trend of incidence, sub-site distribution and staging of colorectal neoplasms in the 15-year experience of a specialized cancer registry Ann Oncol 2004 15 940 946 10.1093/annonc/mdh224 15151952

[B39] Abdulkareem FB Abudu EK Awolola NA Elesha SO Rotimi O Akinde OR Atoyebi AO Adesanya AA Daramola AO Banjo AAF Anunobi CC Colorectal carcinoma in Lagos and Sagamu, Southwest Nigeria: A histopathological review World J Gastroenterol 2008 14 6531 6535 10.3748/wjg.14.6531 19030207 PMC2773341

[B40] Hamilton SR Bosman FT Boffetta P Bosman FT, Carneiro F, Hruban RH, Theise ND Carcinoma of the colon and rectum WHO Classification of Tumors of the Digestive System 2010 Lyon: IARC Press 134 146

[B41] Missaoui N Jaidaine L BenAbdelkader A Beizig N Anjorin A Yaacoubi MT Hmissa S Clinicopathological patterns of colorectal cancer in Tunisia Asian Pacific J Cancer Prev 2010 11 1719 1722 21338221

[B42] Ashktorab H Smoot DT Carethers JM Rahmanian M Kittles R Vosganian G Doura M Nidhiry E Naab T Momen B Shakhani S Giardiello FM High incidence of microsatellite instability in colorectal cancer from African Americans Clin Cancer Res 2003 9 1112 1117 12631615

[B43] El-Bolkainy TN Sakr MA Nouh AA El-Din NH A comparative study of rectal and colonic carcinoma: demographic, pathologic and TNM staging analysis J Egypt Natl Canc Inst 2006 18 258 263 17671536

[B44] Elesha SO Owonikoko TK Colorectal neoplasms: a retrospective study East Afr Med J 1998 75 718 723 10065214

[B45] Winawer SJ Zauber AG Ho MN O'Brien MJ Gottlieb LS Sternberg SS Waye JD Schapiro M Bond JH Panish JF Prevention of colorectal cancer by colonoscopic polypectomy N Engl J Med 1993 329 1977 1981 10.1056/NEJM199312303292701 8247072

[B46] Overman MJ Hoff PM EGFR-targeted therapies in colorectal cancer Dis Colon Rectum 2007 50 1259 1270 10.1007/s10350-007-0228-3 17566832

[B47] Datubo JCB Naaeder SB Tettey Gyasi RK Colorectal carcinoma: an update of current trends in Accra WAJM 2010 29 178 183 10.4314/wajm.v29i3.68218 20665462

